# Mental health workers’ perspectives on peer support in high-, middle- and low income settings: a focus group study

**DOI:** 10.1186/s12888-022-04206-5

**Published:** 2022-09-10

**Authors:** Silvia Krumm, Maria Haun, Selina Hiller, Ashleigh Charles, Jasmine Kalha, Jackie Niwemuhwezi, Rebecca Nixdorf, Bernd Puschner, Grace Ryan, Donat Shamba, Paula Garber Epstein, Galia Moran

**Affiliations:** 1 Department of Psychiatry and Psychotherapy II, Ulm University, Ulm/Guenzburg, Germany; 2 School of Health Sciences, Institute of Mental Health, University of Nottingham, Nottingham, UK; 3 Centre for Mental Health Law and Policy, Indian Law Society, Pune, India; 4 Butabika National Referral Hospital, Kampala, Uganda; 5 Department of Psychiatry and Psychotherapy, University Medical Center Hamburg-Eppendorf, Hamburg, Germany; 6 Centre for Global Mental Health, London School of Hygiene and Tropical Medicine, London, UK; 7 Department of Health Systems, Impact Evaluation and Policy, Ifakara Health Institute, Dar es Salaam, Tanzania; 8 Bob Shapell School of Social Work, Tel Aviv University, Tel Aviv, Israel; 9 Department of Social Work, Ben Gurion University of the Negev, Be’er Sheva, Israel

**Keywords:** Peer support, Mental health workers, High-, middle- and low income settings, Focus groups

## Abstract

**Background:**

Peer support is increasingly acknowledged as an integral part of mental health services around the world. However, most research on peer support comes from high-income countries, with little attention to similarities and differences between different settings and how these affect implementation. Mental health workers have an important role to play in integrating formal peer support into statutory services, and their attitudes toward peer support can represent either a barrier to or facilitator of successful implementation. Thus, this study investigates mental health workers’ attitudes toward peer support across a range of high- (Germany, Israel), middle- (India), and low-income country (Tanzania, Uganda) settings.

**Methods:**

Six focus groups were conducted in Ulm and Hamburg (Germany), Butabika (Uganda), Dar es Salaam (Tanzania), Be’er Sheva (Israel), and Ahmedabad, Gujarat (India) with a total of 35 participants. Transcripts were analyzed using thematic content analysis.

**Results:**

Participants across the study sites demonstrated overall positive attitudes towards peer support in mental health care, although some concerns were raised on potentially harmful effects of peer support such as negative role modelling and giving inadequate advice to service users. Notably, mental health workers from low- and middle-income countries described peer support workers as bridge-builders and emphasized the mutual benefits of peer support. Mental health workers’ views on peer support workers’ roles and role boundaries differed between sites. In some settings, mental health workers strongly agreed on the need for role clarity, whereas in others, mental health workers expressed mixed views, with some preferring blurred role boundaries. Regarding collaboration, mental health workers described peer support workers as supporters and utilizers, equal partners or emphasized a need for trust and commitment.

**Conclusions:**

Mental health workers’ attitudes toward peer support workers were positive overall, but they also varied depending on local context, resources and previous experiences with peer support. This affected their conceptions of peer support workers’ roles, role clarity, and collaboration. This study demonstrated that reconciling the need for local adaptations and safeguarding the core values of peer support is necessary and possible, especially when the implementation of recovery-oriented interventions such as peer support is accelerating worldwide.

**Supplementary Information:**

The online version contains supplementary material available at 10.1186/s12888-022-04206-5.

## Background

Peer provided support for service users is increasingly acknowledged as an integral part of the mental health care systems. Supported by empirical evidence for equal or better outcomes for service users [1–4], numerous peer support programs have been implemented in a variety of different mental health services [5, 6]. However, most research on peer support comes from high-income countries (HIC) in North America, Australia and Europe. Less is known about the implementation and effectiveness of peer support in low- and middle-income countries (LMICs) [1, 2, 6]. Researchers in global health generally (and global mental health specifically) advocate for implementation science to investigate interventions like peer support in the contexts in which they take place, documenting local adaptations and use as well as responsiveness to the local needs of the community [7, 8]. Assessing the contextual factors that impact peer support in different settings also enables the identification of underlying mechanisms for successful implementation, offering a fuller picture and deeper understanding of how peer support works [9].

Successful implementation of peer support in mental health services depends on several factors. A recent systematic review of 53 studies has identified eight key factors, each with the potential to facilitate or impede the implementation of peer support, comprising cultural, organizational and structural aspects, peer training, role definitions, and support for peer support workers [10]. Mental health workers (MHWs) can have a major influence on these factors. For example, MHWs may facilitate peer support by giving positive responses to peer support workers, by having sufficient knowledge to hire peer support workers (PSWs) and to give advice on integrating the PSWs’ role. On the other hand, where MHWs have less experience with PSWs and less understanding of the PSWs’ role, negative attitudes and uncertainties about how to interact with PSWs may serve as barriers [10].

There is evidence for MHWs’ overall positive assessment of peer support interventions [11–13]. However, some MHWs perceive peer support models as threatening to their own roles and responsibilities [12, 14, 15]. Role confusion between PSWs and service users has also been identified as a key barrier to successful implementation of peer support, and role clarity is emphasized in order to avoid tokenism [11, 12, 15, 16]. Although several studies have explored MHWs’ attitudes towards peer support, this research has been done exclusively in higher-income settings, such as in the US [17, 18], Europe [11–13, 15, 19–22] and Australia [23, 24] with questionable transferability of findings across different countries and settings. To the best of our knowledge, no empirical evidence from a comparative analysis of MHWs’ attitudes on peer support in different settings is available.

In order to address this gap, we conducted a comparative investigation of MHWs’ perspectives towards peer support across different mental health settings in LMICs and HICs. By taking into account the local context for the provision of peer support, we aim to develop a deeper understanding of MHWs’ attitudes and experiences with PSWs across different settings and thus, an enhanced understanding of the peer support concept.

This study is part of the UPSIDES project (‘Using Peer Support In Developing Empowering Mental Health Services’). UPSIDES is an international multicenter study which aims at scaling-up peer support for people with severe mental illness in high-, middle-, and low-income countries including Germany, Uganda, Tanzania, Israel, and India through mixed-methods implementation research [25]. Severe mental illness was defined as ≥5 points on the Threshold Assessment Grid, TAG [26] and illness duration ≥2 years. In addition to the TAG and duration criterion, PSWs in each site are expected to work with appreciable numbers of individuals with e.g. psychotic features, mood problems, and anxiety/trauma related problems.

UPSIDES PSWs are people with lived experience of mental health problems who were trained to utilize their own experiences to help facilitate, guide and mentor their client’s recovery journey. The training manual consists of 12 modules including e.g. recovery planning, communication or role description [26]. Training on local adaptations is provided via additional modules that consider context and address site-specific topics (e.g. resources in mental health care or stigma). PSWs receive regular supervision provided by a mental health professional and/or an experienced colleague and intervision (group sessions of the PSW team moderated by a mental health professional and/or an experienced colleague). UPSIDES peer support is delivered for up to 6 months, with a minimum of 3 contacts with the service user [27]. See Nixdorf et al. (2022) for further details on the intervention [28].

In addition to a pragmatic randomized controlled trial assessing the effectiveness of UPSIDES peer support, evaluation of the intervention includes a qualitative study of the subjective perspectives of different stakeholders, before and after implementation [27].

The qualitative study includes focus groups with MHWs (e.g. nurses, psychiatrists/physicians or psychologists) at each study site prior to the implementation of UPSIDES peer support, addressing the following research questions:

1. What are MHWs’ expectations on PSWs’ roles and responsibilities?
2. What are MHWs’ views on challenges in working with PSWs?
3. What are MHWs’ expectations on collaboration between MHWs and PSWs?

## Methods

We followed COREQ guidelines for reporting on qualitative studies [29].

### Study sites

The study took place at the six study sites Ulm and Hamburg (Germany), Butabika (Uganda), Dar es Salaam (Tanzania), Be’er Sheva (Israel), and Ahmedabad, Gujarat (India). The study sites differed in terms of service provision, urban vs. rural setting, experiences with PSWs, and payment of PSWs [10, 27]. A summary of the study context can be found in the Supplementary Table (see Additional file 1).

### Recruitment

Study participants were recruited from multidisciplinary mental health teams including members from different professional disciplines, e.g. psychiatrists, psychotherapists, nurses, social workers. Research workers at each site used purposive sampling strategies in order to reach potential participants. Inclusion criterion was that MHWs were expected to work together with PSWs during the UPSIDES intervention. Potential participants were contacted in person, by email or by phone. In addition, the study aims and focus group procedures were introduced by local meetings (e.g. clinic conferences and local advisory board meetings). MHWs interested in study participation received an invitation (via a written letter or e-mail) with specific dates and venue. For their participation, participants received a small financial allowance, depending on the study site-based policies.

### Focus groups / participants

At each study site, one focus group with MHWs was conducted resulting in a total of six focus groups. The focus groups were conducted prior to the implementation of UPSIDES peer support between Nov. 2019 and Jan 2020. The focus groups took place in mental health service settings and lasted between 45 and 120 min. More than half of the participants were female (20 out of 35). The MHWs’ average age was 37 years with a range of 22-63. The majority of the participants had at least some experiences with peer support.

Table 1 shows the focus group characteristics of the focus groups and study participants.

**Table 1:** Characteristics of focus groups and study participants

Study Site	BGU	BU	DS	AD	UKE	Ulm
N	5	6	7	4	5	8
Mean Age (range)	33 (25-39)	38 (31-48)	36 (29-55)	38 (28-51)	35 (22-63)	45 (27-55)
Gender	f:3; m:2	f:3; m:3	f:4; m:3	f:1; m:3	f:4; m:1	f:5; m:3
MHWs’ Professional Background	Rehabilitation coordinator; Social worker - coordinator; Employment Accompaniment; Social worker - rehabilitation coordinator ; Social worker - rehabilitation coordinator	Nursing Officer; Nursing Officer; Psychiatric clinical officer; Psychiatric clinical officer; Social worker; Occupational therapist	Health social worker; Registered nurse; Social worker; Health social worker; Assistant nursing officer; Nursing officer; Nursing officer	Medical Officer; Psychiatric Nurse; Psychiatric Social Worker; Psychiatrist	Peer Support Worker; Psychologist and trainee therapist; Psychologist and research assistant; Student of psychology; Internship student of psychology (research assistant)	Medical doctor; Medical doctor; Psychologist; Psychologist; Psychologist; Care giver; Care giver; Pedagogue
MHWs’ experiences with PSWs	Yes:5; No:0	Yes:3; No:3	Yes:4; No:3	Yes:3; No:1	Yes:4; No:1	Yes:6; No:2
Date	December 22, 2019	January 9, 2020	January 10, 2020	November 26, 2019	January 17, 2020	November 26, 2019
Duration (min)	90	120	45	67	61	62

*BGU* Be′er Sheva (Israel), *BU* Butabika (Uganda), *DS* Dar es Salaam (Tanzania), *AD* Ahmedabad (India), *UKE* Hamburg (Germany), *Ulm* Ulm/Guenzburg (Germany)

*MHWs* Mental Health Workers, *PSWs* Peer Support Workers

### Data collection

Data were collected using a semi-structured topic guide. The topic guide was developed in cooperation between task leads and partners at each study site. The task leads (SK, MH, GM, and PGE) provided a preliminary topic guide guided by the Consolidated Framework for Implementation Research (CFIR) [30] and evidence-based literature [10]. Research workers at each site reviewed the topic guide. The topic guide included the following 3 topics: 1) collaboration with PSWs, 2) organizational and team culture, 3) needs for support. Each topic was introduced by a key question followed by several sub questions. Moderators asked sub questions if the key question did not lead to the emergence of sufficient content during the discussion of particular topics. The semi-structured format of the topic guide allowed flexibility in order to capture “new” or locally relevant aspects. The focus group guide can be found in the Supplementary Table (see Additional File 2).

A short questionnaire was given to participants. The questionnaire gathered basic demographic information including participants’ gender, professional background, years of professional experience, and experiences with peer support work (yes/no).

Focus group moderators at each site received study-specific online training on qualitative research and written and/or verbal instructions on how to conduct the focus group in order to enhance comparability. Instructions included prompts for the introduction, adherence to the topic guide, dealing with additional comments, managing potential problems, e.g. participant’s lack of response, and guidelines for moderator’s performance before, during and after the focus groups. The focus groups took place in a conducive environment in line with local expectations around research hospitality (e.g. drinks/snacks provided) at a place, date and time convenient for the participants (Table 1). Each focus group session was facilitated by a moderator and an assistant. The assistant provided technical support. Focus groups were conducted in the local language or English (Kampala) and were audio recorded. After each session, the moderator and assistant documented their initial reflective thoughts on the main topics, impressions on the dynamics of interactions, and parts of discussions that produced rich conversations in relation to the research question.

### Transcription and translation

Study materials including topic guides were forward translated from English to the local language (except Butabika). Audio files were transcribed verbatim in the language used in the focus group sessions. Transcripts were back checked by the moderator against the recording for accuracy. Personal information in the transcripts was deleted or replaced with participant numbers (e.g. mental health worker 1). Forward translation was chosen to maximise feasibility without losing meaningful semantic content. The translation was performed by a bilingual speaker at each site. The translation aimed at conceptual equivalence allowing adaptations to the local context by rewording some questions. In order to ensure consistent analysis across sites, the bilingual speaker translated the transcripts and field notes from the local language into English before finalization. Two researchers as part of the UPSIDES translation team checked all final back-translated transcripts and field notes to ensure comprehensibility for analysis.

### Analysis

Focus group data were analyzed thematically by a core group of researchers (MH, SK, GM, PGE). Following the approach of Braun and Clarke [31], the analysis took place using three steps. In step one, two transcripts were consecutively read several times by two independent research workers and preliminary codes and themes were developed and reviewed by the core group of analysists. In step two, the preliminary coding-tree created through the analysis of the two transcripts was then applied to the remaining four transcripts, and further codes were consecutively added to the coding tree. In step three, themes were reviewed, refined, modified and structured until a consensus on the final coding tree was reached. The process of data analysis and the formation of preliminary codes and themes was critically discussed during two qualitative research workshops. In case of uncertainties or difficulties in understanding arising from the analysis of transcripts, a research worker from the relevant site was consulted. In order to enhance rigor, a simplified coding tree with example quotes was discussed during the qualitative research workshop at the Ulm study site. The finalized version was then introduced to the research workers at each site. Minor changes relating to the coding of certain quotes were made according to the feedback from one site. Triangulation was carried out by the use of multiple analysts to ensure that the interpretations were informed by a range of perspectives (investigator triangulation), the analysis was data centered (interpretations close to data), the verbatim quotes that underlie interpretations were transparent, and the codes were validated through consultation with local research teams. We used MAXQDA 2011 and MAXQDA 2020 for managing codes, themes and memos. Memos were written throughout the analysis process to capture thoughts and reflections about MHWs' statements.

## Results

Based on codes derived from the topics 1. “Collaboration” and 2. “Organizational and team culture”, we identified four main themes to describe MHWs’ views on 1) expected benefits from peer support, 2) challenges and concerns about risks of peer support, 3) roles and boundaries and 4) team collaboration and PSWs’ position in mental health services. The findings derived from the topic 3. “Support” will be published in a separate paper dealing with MHWs’ expectations towards and recommendations on the implementation of peer support. Table 2 summarizes the themes and subthemes. *(Insert* Table 2*: Themes).*

**Table 2:** Themes and subthemes

Main Themes	Subthemes I	Subthemes II
1. Expected benefits from peer support	Benefits for service users	Supporting service users through sharing lived experiences
		Supporting service users through (in)formal arrangements
		Supporting service users in their communities: bridge building
	Benefits for PSWs	
	Benefits for MHWs and mental health services	
2. Challenges and concerns about peer support	Negative effects on service users	Negative role model
		Knowledge, skills and training
	Negative effects on PSWs	
	Negative effects on MHWs teams and institutions	
3. PSWs’ roles and boundaries	(Need for) role clarity of PSWs	
	Acceptance of role ambiguities	
	“New” vs. “established” PSWs’ roles	
4. Team collaboration and PSWs’ position in mental health services	PSWs as supporters and utilizers of the mental health system	
	PSWs as “equal” partners	
	Collaboration as (controlled) trust and commitment	

*MHWs* Mental Health Workers, *PSWs* Peer Support Workers

Themes are partially illustrated by participant quotes. Further illustrating quotes can be found in the Supplementary Text (see Additional file 3). Participant quotes are labelled according to the study site Ulm (Ulm), Hamburg (UKE), Butabika (BU), Dar es Salaam (DS), Be’er Sheva (BGU), and Ahmedabad, Gujarat (AD) and the transcript chapter.

### Expected benefits from peer support

Participants discussed a broad range of positive expectations from PSWs including benefits for service users, benefits for PSWs, and benefits for mental health institution.

Participants discussed benefits for service users in terms of sharing experiences, bridging, and informal/formal arrangements. Supporting service users by sharing PSWs’ lived experiences was a dominant theme throughout all focus group discussions. At the same time, the topic of PSWs’ contributions regarding formal arrangements and bridging was particularly discussed in Butabika, Dar es Salaam and Ahmedabad.

Sharing personal experiences was assessed as the key element of peer support and participants often referred to its empowering function. PSWs were described as role models for service users in their recovery processes by providing hope for a positive future and promoting service users’ recovery oriented attitudes:

“*Live positively with mental illness and also to take care of themselves in that even when they leave the hospital, they can also be home and be very productive, other than being dependent on people around them or thinking they can only survive on the hospital as most patients believe”* (*BU 11*).

*“It comes from this idea to say, you have a shared experience and you make use of it and you get into a real contact and a real conversation and get to know each other and see what new possibilities I have? What do I actually want for my life? How can I get there? This is, I think, something I can’t really tell the difference between peer support in general”* (*UKE 57*).

Sharing personal experiences was frequently valued as a unique and authentic contribution from PSWs. Compared to the often hierarchical power relations in traditional mental health services, PSWs are able to build more balanced partnerships and thus, enjoy more credibility from service users (Ulm 90) (UKE 186).

*“Earlier we used to advise the patient that you should do this or that. In contrast, a PSW makes the patient understand that they have also been affected by this disease and have recovered (…) I’m here for you, I support you, tell me what is going on, what do you want to do such that you improve, in contrast to advice”* (*AD7*).

*(PSW use) “a different language than staff members (…) can reach service users on a different or common sense level and not that much on a professional one (…) PSW are allowed to say things directly and frankly because it’s more on an eye-to-eye level”* (*Ulm 86*).

*(PSWs) “can easily explain how an experience is and how to live positively more than a mere mental health worker who has never experienced that” *(*BU 23*).

This unique relationship, in turn, makes it easier for service users to accept help and support.

*“Kind of ‘door opener’ for very sceptical patients with strong reservations against psychiatry, PSW can share their experiences and allay fears (…) because in acute situation there is often mistrust against staff”* (*Ulm 108*).

“*Let’s say, in a group I am facilitating, so we’ll say the same sentence. It will sound different if I or my partner say the same thing. Hmm… it’s not always that dramatic, it doesn’t always make a difference, but there are sentences, that if she says and if I say (makes a difference, SK)*” (*BGU 97*).

Besides personal support through lived experiences, participants emphasized PSWs’ contributions in terms of different kinds of formal and informal support and arrangements including education and motivation or helping with administrative issues. Participants in Dar es Salaam described how PSWs can educate clients to develop a lifestyle which decreases the risk for relapse (DS 12).

Supporting clients with medication was an important issue that emerged in Dar es Salaam and Butabika. Participants expected PSWs to encourage clients to take their medication and to follow MHWs’ instructions around medical treatment including both medical side effects and the risks of medication non-compliance. From such views, PSWs' personal experiences can complement MHWs’ expertise on medication treatment:

*“We will tell them about the side effects of the medicine but the peer support worker will be an advocate to give evidence of the side effects in using the medicine and what happened when he didn’t use medication. I will base on the profession part and for them it will (be) evidence based”* (*DS 27*).

In Ahmedabad, PSWs were expected to guide service users through complex administrative processes including admission, assistance in the patient-doctor-talks, and explanations of rules and regulations (AD19, AD27, AD 66, AD 83, AD 137). Helping service users to have their documents ready and available has the potential to facilitate admission processes (AD 19). PSWs were also expected to gather important information on the service users:

*“Observing them, knowing who’s their relatives, education level, taking initiative and talking to them, trying to understand the problem through the patient’s relatives” (AD 19).*

Some participants expected PSWs to be bridge builders between hospital and community settings. The connecting function of PSWs was a discussion point in Butabika and Dar es Salaam, where PSWs provide hospital but also community services (see Supplementary Table, Additional file 1). In Ahmedabad, where peer work is hospital based, participants emphasized PSWs’ role in bridging the gap between service users and the hospital setting:

*“I think the main thing PSV should realize is that they are the link between hospital and the patient (everyone nods yes). They are link between hospital and patient (…) that is what should be developed and PSWs should feel that his role is for making the patient get in touch, not forcefully, with the hospital services” (AD 117).*

Participants in Dar es Salaam (DS 54), Ahmedabad (AD 7) and Butabika (BU 27) emphasized that PSWs play an important role in supporting and enhancing the reintegration of hospital patients into communities, e.g. by engaging relatives of service users:

*“A PSW should also be an ambassador to the service users, he/she should educate them and also engage those people who are close to the service user” (DS 18).*

PSWs are described as often more familiar with local cultures than MHWs and thus, could better serve as bridge builders. By reaching out into communities, PSWs in Dar es Salaam and Butabika were described as increasing local community awareness of mental health issues while also decreasing barriers for community members to access mental health services. From their views, PSWs contribute substantially to the acceptance of mental health services and to the de-stigmatization of mental disorders (BU 27)(DS 54).

Beyond the positive impact of PSWs on the recovery processes of service users, participants also talked about benefits for the PSWs. In Butabika, Dar es Salaam and Ahmedabad MHWs expected that working as a PSW has a positive impact on the personal recovery of the PSWs themselves. Supporting service users enables PSWs to live a *“useful”, “stable”* (BU 19) or *“economically independent”* (AD 244) life in the community. By providing peer support for service users, PSWs learn that *“they are useful in a way that they can be able to support the community so they also have to look after themselves very well to make sure that they are stable”* (BU 19). Helping service users to cope with daily challenges can have a positive effect on the recovery process of the PSWs (DS 25). From participants’ perspectives, PSWs can themselves benefit from the close relationship with mental health staff and from the provision of services and resources including medication (DS 20).

Finally, focus group participants discussed benefits for the mental health services. From this perspective, MHWs themselves can learn from PSWs as role models and from shared experiences:

*“To me that is inspiration, to see a woman who went through so much, still going through so much, and still there is something very powerful about her, very strong* “*(BGU 171).*

*“It’s a two-way opportunity in here. Working with a PSW helps a mental health worker to learn about the challenges in terms of treatment and recovery” (BU 27).*

Participants in Be’er Sheva expected a positive impact from the UPSIDES peer support on the mental health team (BGU 113). In Dar es Salaam and Ulm, the work of PSWs is valued because PSWs can ease MHWs’ workload in regard with health education (DS 77)(Ulm 102).

### Challenges and concerns about peer support

Generally, the MHWs’ views of PSWs depend on individual attitudes and experiences with PSWs. This includes a broad spectrum of many positive to some negative appraisals too. These complexities and ambivalences in MHWs’ attitudes were addressed in Be’er Sheva (BGU 114). Throughout all focus groups, participants shared concerns regarding the successful implementation of peer support including the negative effects of peer support on the service users, on the PSWs themselves and on the quality and reputation of the mental health institution.

Focus group participants discussed negative effects on service users in regard with PSWs serving as negative role models and PSWs’ limited knowledge, skills and training. In contrast to the impact of PSWs being a positive role model, participants raised concerns about PSWs as “negative” role models. Some participants addressed demotivating effects from negative role models, e.g. for younger and/or first time service users. Referring to experiences with a PSW with long-term experiences as a service user, participants in Ulm expressed such concerns: PSWs’ identification with *“chronic”* service users might counteract with the aspirations of younger, first-time service users to return to a *“normal”* life (Ulm 127)(Ulm 237). In Butabika, concerns were raised on the negative effects on service users when PSWs experience relapses while providing peer support (BU 39). One participant in Be’er Sheva was concerned that service users could be harmed or even traumatized by PSWs sharing their experiences with service users (BGU 73).

Closely linked to the before mentioned risks, doubts were raised on PSWs’ knowledge and qualifications in relation to professionally trained staff. Participants in Ulm expressed fears that PSWs without adequate training, skills and knowledge about specific therapeutic concepts may thwart the delivery of therapy and thus, exert a negative impact on service users (Ulm 136). Participants in Ulm and Dar es Salaam shared sceptical views on the risk of *“inadequate recommendations”* particularly with regard to medication and rehabilitation aims (DS 37)(DS 38)(Ulm 49)(Ulm 125)(Ulm 126). Against the background of the risks relating to disclosing and sharing PSWs’ own experiences, participants in Be’er Sheva emphasized that PSWs should have developed specific skills and undergone their own recovery process before providing peer support (BGU 150).

Besides the positive impact from providing peer support, some participants discussed the risk of negative effects of peer support for the PSWs themselves (BGU 129). PSWs are described as vulnerable and thus, sometimes incapable to endure “usual” workplace stress. A participant in Ulm referred to earlier experiences with a PSW who was burdened by a team conflict (Ulm 259):

*“That somehow decompensated him and I know that he was going to hospital one week later. (…) well, (peer support work) should also be something supportive for the peer and something that promotes self-esteem and not become a burden and I had the feeling that (PSW) put himself through too much and in the end, it was a burden for him too”* (Ulm 259).

Financial reimbursement was an issue. Depending on the payment system for PSWs, participants stressed that PSWs should be paid better. Participants also mentioned the long-term effects for PSWs’ work rehabilitation in terms of being stuck in a low paid job without any opportunities for a better-paid job outside mental health settings (AD 303)(UKE 17).

In particular, participants in Ulm and Dar es Salaam expressed some fears on service users receiving “unqualified” services by PSWs which could put damage on the relationships with service users and on the reputation of the mental health institution as a whole (DS 37).

*“They just go into a one-on-one conversation after a short training and who knows what they are talking about with them. And we have no control over that at all. Of course, this always happens when patients talk to each other, e.g. not taking medications anymore. But I just think that if it is an “official” service then, this is just not…it has no quality and we have to control that in any case and I think it is very important that they really get sufficient training and supervision”* (Ulm 125).

A participant in Be’er Sheva expressed fears of being overburdened by working alongside PSWs (BGU 129).

### PSWs’ roles and boundaries

A considerable part of the focus group discussions related to issues regarding roles and cooperation between MHWs and PSWs including role boundaries, responsibilities, and formal structures of cooperation. In Ahmedabad, Dar es Salaam, Butabika and Ulm, participants stressed a need for role clarity. In contrast, some participants in Be’er Sheva tended to accept blurred role boundaries or role ambiguities. Participants in Hamburg reported difficulties with “new” vs. “established” PSWs’ roles.

In Ahmedabad, Dar es Salaam, Butabika and Ulm, MHWs’ need for PSWs’ role clarity was evident. Participants shared a widely unquestioned understanding of PSWs’ duties and tasks that were seen in clear contrast to the roles of MHWs based on formal education and professional knowledge (DS 33)(AD 425).

*“It’s important to know their limits because sometimes they might cross over to do roles that are not intended within the domain of peer support and might go further to do the medical work” (BU 40).*

While emphasizing the importance of role clarity between MHWs and PSWs, such clarity was assessed as not reached yet. This was apparent when the focus groups discussion started with a number of open questions relating to PSWs’ roles and a strong agreement on the need for clarity (Ulm 25). Participants in Ulm, Butabika, Ahmedabad and Dar es Salaam expressed a strong need for clear roles regarding responsibilities, access to (confidential) information and respecting boundaries in the therapeutic relationships between PSWs and service users. It was emphasized that PSWs should critically reflect their personal relations with the service users including physical contacts:

“*How do we deal with this friendly contact with the patient (…) physical contact with the patient (…) in retrospect we hear that this was actually too close and too uncomfortable. Such things should be discussed with the staff in advance. Where’s the limit? What am I allowed to do in my work, what am I not allowed to do?” (ULM 69).*

In line with this, participants reported that they were afraid of role ambiguities because of the inherent risk of dissolution of given structures (Ulm 29). Some participants raised concerns on the blurred lines and interchangeable characteristics of PSWs’ roles between being a “bridge builder” and being a (former) service user with mental health needs (AD 109). Similarly, participants in Ulm reported uncertainties on how to deal with (acute) mental health needs of PSWs. Some participants were sceptical about the inclusion of PSWs into teams where they were formerly treated by the MHW team. In another statement, a participant referred to the need for distinguishing between MHWs and service users - with the risk of neglecting PSWs’ mental health needs (Ulm 318).

Role confusion was assessed not only as a risk factor for the PSWs themselves. Participants in Ahmedabad expressed fears of a negative impact of role uncertainty for the peer support concept.

*“But the important thing is that if they (PSWs) are also considered professionals then the peer feeling will go away”* (*AD 429*).

In contrast to widely shared views on role boundaries between MHWs and PSWs and a strong need for role clarity, the role issue was discussed controversially in Be’er Sheva (BGU 171). Although not all participants agreed with such a perspective, some provided strong arguments for the existence of similarities between PSWs and MHWs regarding their mental health experiences*:*

*“There is no difference between someone who is a professional employee and someone who is a consumer-as-provider (…) we all come from a certain life experience, with certain difficulties that we encounter, facing the service-users and we really all work on it. With him, certain problems will come up, and with him, certain problems will come up” (BGU 179).*

However, some participants also reported problems that could emerge from role diffusions, for example when consumers as providers  do not share their knowledge about service users with MHWs (BGU 73). Therefore, for a few participants, role definitions are sometimes necessary (BGU 167).

On account of the long-standing experiences with peer support services, participants in Hamburg did not discuss PSWs’ roles and responsibilities in relation to those of MHWs. Rather, the focus groups turned into a controversial discussion on the “new” vs. “established” PSWs and their roles. Participants raised concerns on how to match the “new” UPSIDES intervention with an established program. UPSIDES was assessed as a competing program to an established peer support program (“Ex-In”):

*“B1: What irritates me is also () I can already see from the offer that it is a definite competition.*

*I: It’s not supposed to be there.*

*B1: It’s not supposed to be that, I already said that, we talked about it a lot. But the feeling is just there. Where is there a difference?” (UKE 35).*

As a consequence of the competing structure of PSWs, participants discussed several risks including decreasing recognition of the established peer program, qualification of PSWs, unequal payment as a result of different program financing, and different PSWs’ tasks due to time and costs of the two (different) training programs.

### Team collaboration and PSWs’ position in mental health services

Focus group participants discussed different forms of collaboration between MHWs and PSWs and positions of PSWs in the mental health services including PSWs as supporters and utilizers of the mental health system, PSWs as “equal” partners, and collaboration as (controlled) trust and commitment.

MHWs’ perspectives on PSW’s roles and responsibilities were closely related to their views on collaboration options and the assigned status of PSWs in the mental health service system. Participants in Butabika, Dar es Salaam and Ahmedabad described PSWs as supporters of the mental health system including a medical focus on mental health. MHWs mentioned mutual benefits of collaborations with PSWs: On the one hand, mental health services benefit from peer support (BU 33)(DS 77). On the other hand, participants expressed caring attitudes towards PSWs (DS 31)(DS 76)(AD 300). For participants in Ahmedabad, as a consequence of the mutual benefit, anticipated risks and challenges for PSWs can be managed by guidance and support by MHWs (AD 147).

Participants in Be’er Sheva shared an understanding of an (ideal of) equal partnership between MHWs and PSWs *(BGU 113).* They emphasized a strong orientation towards cooperating at eye-level where both bring a distinct but equally valuable experience. From this perspective, PSWs and MHWs complement each other equally and contribute “as partners” (BGU 70)(BGU 91). Consequently, participants expressed accepting attitudes including approaches focusing on capability, empowerment and strengths, even during challenging situations (BGU 66). Mutuality of trust and exchange between team colleagues was emphasized (BGU 70). Participants highlighted the “flexibility” of mental health services as an important prerequisite for implementing peer support programs and for delivering of peer support (BGU 133). However, stigma was mentioned as an important barrier to the acceptance of PSWs from MHWs and service users as well. References to stigma mirror hierarchical structures where PSWs are perceived as being in a “lower” status (BGU 38)(BGU 114).

From the focus group discussion in Hamburg, PSWs appear as an integral part of mental health teams. This is evident from both explicit reports and implicit focus group dynamics as well. Although each study site was asked to form their focus group with MHWs only, the focus group in Hamburg consisted MHWs and a PSW as well. Obviously, it seemed self-evident to include PSWs in a focus group with MHWs. In addition, participants explicitly referred to the positive developments relating to the collaboration between “established” PSWs and mental health teams resulting in a “grown together” attitude:

*“Meanwhile it’s growing together really well, because we notice that we complement each other really well” (UKE 91).*

Although generally appreciating the concept of peer support, the attitudes of the participants in Ulm were influenced by earlier and some negative experiences with peer support work. The main concerns related to insufficient knowledge on PSWs’ knowledge, roles and responsibilities, and participants emphasized the need for role definition and transparency on peer support program and training. Open exchanges about PSWs’ tasks or responsibilities were seen as key requirements for successful implementation of peer support. In their views, *“structural order and transparency”* (Ulm 218) and personell continuity (Ulm 214) are the foundation for successful collaboration and fostering trust and commitment (Ulm 214)(Ulm 218)(Ulm 237).

*“Regarding collaboration, it is very important to have good exchange and open discussion. I don’t want to have the feeling that PSWs are doing their own thing which we do not know anything about it. Openness and feed-back is very very important”* (Ulm 37).

## Discussion

This study provides insight into similarities and differences in MHWs’ attitudes about peer support across six study sites in Africa, Asia and Europe. Most similarities were found in regard with MHWs’ general valuation of peer support. At the same time, MHWs' views differed on a number of issues, including expectations towards tasks and responsibilities of PSWs, attitudes towards PSWs’ roles, and team collaboration. In order to understand MHWs’ attitudes towards PSWs, it is essential to consider the local context in terms of the socioeconomic conditions, former experiences with PSWs and the embeddedness of peer support in organizational cultures. While most peer support programs have their roots in Western service user activist movements, there might be different context conditions for peer support in other settings [14]. The 6 study sites varied in their previous experiences with peer support [10]. While for some study sites, peer support is a more recent development and not closely linked to an established service users’ activist movement, others look back to longer traditions. In India, peer support in mental health care has been provided since 2015, while Uganda started in 2011 with particular peer support programs supported by a consumer organization [32–34]. Peer support in Tanzania was delivered in HIV care but not in the context of mental health treatment [35]. Israel and some parts in Germany are characterized by a longer tradition in recovery oriented services including peer support (Hamburg) or consumers-as-providers (Be’er Sheva) programs, and a strong interconnection with service users activist movements [36, 37]. At the study site in Ulm (Germany), peer support is a relatively new intervention and not linked to a local service users’ activist movement.

Our findings give further evidence to MHWs’ generally positive attitudes towards peer support in adult mental health services [11–13]. Participants across all study sites valued peer support for a number of benefits for service users, PSWs, and the mental health services. Participants emphasized PSWs’ positive impact on service users’ recovery-oriented processes through sharing lived experiences and positive role models, and the expectation of less hierarchical relationships between service users and PSWs that make it easier for service users to accept help. This is consistent with other findings on MHWs’ perspectives towards PSWs having less professional distance towards service users, and on PSWs’ serving as a “bridge” between service users and MHWs leading to improved adherence and recovery [19]. Particularly, participants in Ahmedabad, Dar es Salaam and Butabika emphasized the importance of PSWs’ role as “bridge builders” between hospital and communities, respectively between hospital and patients. By building bridges between mental health services and local communities, PSWs can promote mental health awareness, reduce stigma and discrimination, support recovery and social inclusion, prevent mental disorders and protect human rights [38].

PSWs were explicitly valued for their contributions to decrease MHWs’ workload in the provision of mental health services. On a global level, peer support is described as a promising approach to overcome access barriers due to limited resources for mental health care [14, 39]. There is a risk, however, that PSWs, who often receive little payment, could be the “cheaper option” by filling gaps for underfunded services in mental health system [11, 14, 15]. In our study, MHWs described PSWs as both supporters and utilizers: On the one hand, PSWs being employed contributed to their own recovery in settings with limited resources for mental health services including free access to education and/or medication. On the other hand, PSWs play a key role in the provision of mental health services and they may be required to step in for mental health services otherwise not fully accessible due to lacking resources, e.g. informal support during hospital admission. Thus, particularly in settings with limited resources for mental health services, the implementation of peer support might be facilitated by the mutuality of benefits in regard with limited resources.

Concerns about potentially harmful effects of peer support on service users were closely linked to MHWs’ insecurities about PSWs’ responsibilities and roles. Most uncertainties were raised in a setting with less and rather mixed (positive and negative) peer support experiences. This is congruent with other studies investigating MHWs’ perspectives in an early phase of peer support implementation or services with very few PSWs [11–13, 15, 24]. Participants in Ulm perceived the UPSIDES peer support as a new intervention implemented as a top-down process and not linked to a local service users' activist movement. In addition to generally positive attitudes towards the peer support concept, the participants posed a number of open questions and highlighted challenges including a strong call for clarity and transparency. Uncertainties in combination with PSWs’ cheaper workforces might contribute to MHWs’ perception of their roles and responsibilities being threatened by new peer support models [12, 14]. This echoes worries on the challenges relating to top-down implementation of recovery-oriented interventions such as peer support [39].

The lack of role clarity and concerns on role boundaries were often reported as challenges of peer support interventions by both MHWs and PSWs as well [15, 16, 40]. In our study, the existence of and need for clear role descriptions was a controversial issue. Focus group participants in Ulm, Butabika, Ahmedabad, and Dar es Salaam described PSWs’ roles and responsibilities as clearly distinguished from MHWs’ professional roles and they agreed on the need for role clarity as an essential pre-condition for working together with PSWs. Compared to settings with longer traditions of peer support combined with strong political activism towards overcoming the medical model, there might be a risk that PSWs in settings with less peer support experiences inherently reinforce the traditional hierarchies within medical systems [32]. PSWs’ adaptations to traditional clinical structures can reproduce the medical model (including medication) and power structures within mental health teams, and between PSWs and service users [41].

Due to their extensive experiences with recovery oriented services including peer support (Hamburg) or consumers-as-providers (Be’er Sheva) programs, the focus group discussions at these study sites went beyond expectations towards the implementation of peer support in general. Here, PSWs were already assessed as an integral part of the mental health teams. Participants in Hamburg emphasized how to combine two concurrent peer support programs. Concurrent relationships between different peer programs are often a result of limited resources for recovery oriented services with the effect of increasing competitions [41]. Participants in Be’er Sheva intensively discussed how to improve existing peer support processes, e.g. by managing self-disclosures. Given that PSWs are being stigmatized even by service users, participants reflect the ambivalent character of PSWs’ disclosures. Dilemmas emerge when PSWs are expected to share their lived experiences while disclosing the peer status is at risk of being stigmatized [42]. Participants in Be’er Sheva and Hamburg did not discuss roles or role boundaries as implementation barriers. Rather, PSWs were described as “equal” partners. This is in line with reflections on the development of peer support concepts in HIC, where service users’ dissatisfaction with existing mental health services provide the rationale for peer support or other recovery oriented services with the aim of a wider transformation of the traditional, hierarchical and medical-focused mental health system [14]. Although this issue was controversially discussed, some participants in Be’er Sheva even seemed to value blurred role boundaries. This supports qualitative findings that a lack of clear role definitions can offer positive effects for PSWs finding their own role definitions [43]. Considering the long traditions in peer support in Be’er Sheva and Hamburg, less hierarchical relations between PSWs and MHWs appear as a consequent step in transforming traditional mental health systems into services where team members work together as equal partners irrespective of sharing lived experiences.

### Limitations

Our findings are based on a small sample of MHWs at six study site. In addition, participants have been recruited via purposive sampling. Thus, our findings may not be representative of MHWs’ general attitudes at the study sites or in UPSIDES countries more broadly. It is also possible that our findings are biased by the participation of MHWs with specific views on peer support programs. Further, it is possible that MHWs’ attitudes towards PSWs and peer support vary by characteristics of the PSWs, their clients, and the intervention sessions. Unfortunately, the pre-intervention design of this study is not suitable to answer this question. Although UPSIDES peer support is not restricted to specific groups of service users with severe mental disorders, it is possible that some service users due to specific characteristics will not receive UPSIDES peer support. As noted in the introduction, PSWs are expected to work with individuals with e.g. psychotic features, mood problems, and anxiety/trauma related problems. Thus, PSWs are expected to work with a broad range of the problems characterized as SMI. A comprehensive process evaluation will report details about how uptake of UPSIDES peer support varied by characteristics of service users, peer support workers, and study sites [27].

Due to our focus on the collective construction of meaning that emerged in the focus groups, individual opinions and particularly those deviating from the group’s opinion could have been neglected. From a methodological perspective, the thematic analysis did not allow for a deeper understanding of MHWs’ perspectives on peer support, particularly in regard with cultural factors. In addition, most focus group discussions were translated from the local language into English without backwards translation. Thus, in addition with culture-bound perspectives of the analysis team members, it is possible that certain cultural aspects affecting MHWs’ attitudes were overlooked. Finally, MHWs’ reports and opinions on peer support do not provide access to the practices of mental health services. In order to gain a deeper understanding of culturally embedded peer support practices, further studies should include observational data less influenced by the potential social desirability of MHWs’ statements.

## Conclusions

Beyond an overall agreement on the manifold positive effects of peer support, mental health workers differed on a number of issues. These differences could be affected by prior experiences with peer support and available resources. In particular, in LMIC, peer support results in mutual benefits, but there could be a risk that it will be implemented as a “cheap option” for underfunded mental health systems. Mental health teams with less previous experience in recovery-oriented services should be provided with sufficient information on peer support and given time to discuss the possibilities and opportunities for the team to define and understand everyone’s roles [44]. However, although clear role descriptions are important, overformalization of peer support might jeopardize the unique relationship between PSWs and service users. In view of an increasing appreciation of recovery-oriented interventions such as peer support worldwide, taking the local context seriously is important to facilitating successful implementation. At the same time, it is essential to maintain the core ideas and values of peer support, which include equity, hope, trust, respect, acceptance, and shared experiences, across different cultures [14]. South-North and North-South learning is vital to achieving this goal, and it is both possible and fruitful, as this study demonstrates.

## Supplementary Information

**Additional file 1.**

**Additional file 2.**

**Additional file 3.**

## Data Availability

All transcript fragments which informed the analysis presented in this publication are included within the paper and its supporting information files. Full transcripts generated during the current study are not publicly available, as they might identify individuals, even though they are anonymized but are available from the corresponding author on reasonable request.

## Abbreviations

HIC: high-income countries
LMICs: low- and middle-income countries
MHWs: mental health workers
PSWs: peer support workers
UPSIDES: ‘Using Peer Support In Developing Empowering Mental Health Services’
CFIR: Consolidated Framework for Implementation Research

## Study sites

Ulm: Ulm/Guenzburg (Germany)
UKE: Hamburg (Germany)
BU: Butabika (Uganda)
DS: Dar es Salaam (Tanzania)
BGU: Be’er Sheva (Israel)
AD: Ahmedabad, Gujarat (India)
