# Supplementary material for: Mental health workers’ perspectives on peer support in high-, middle- and low income settings: a focus group study
Source: BMC Psychiatry. 2022 Sep 10;22:604. doi: 10.1186/s12888-022-04206-5 (PMC9464408; doi:10.1186/s12888-022-04206-5)
Supplement: Supplementary file 1 — Additional file 1. [file 12888_2022_4206_MOESM1_ESM.docx]

**Additional file 1: Context of the study sites**

| **Host study site** | Dar es Salaam, Tanzania | Butabika, Uganda | Pune, India | Be’er Sheva,  Israel | Ulm/  Guenzburg,  Germany | Hamburg-Eppendorf,  Germany |
| --- | --- | --- | --- | --- | --- | --- |
| **Study setting**  **(abbreviation)** | Muhimbili  National Hospital at the Department of Psychiatry  and Mental Health  (DS) | Butabika Hospital, Kampala  (BU) | Hospital for Mental  Health in Ahmedabad, Gujarat  (AD) | Kidum Proyektim Shikumim  (BGU) | Ulm University’s Department of Psychiatry and  Psychotherapy II at  Bezirks-krankenhaus Guenzburg  (Ulm) | University  Medical Centre Hamburg-Eppendorf  (UKE) |
| **PSWs involved in study site prior to UPSIDES°** | No | Yes | Yes | Yes | No/little | Yes |
| **Mental health services in which UPSIDES PSW are involved in°** | In- and outpatient  services | In- and outpatient  services | Inpatient services | Outpatient  services | Outpatient  services | Outpatient services |
| **Area of UPSIDES PSWs°** | Urban area | Mixed area | Urban area | Urban area | Mixed area | Urban area |
| **Payment of PSWs°** | Small expanse allowance | Expanses for food and travel costs | Regular payment | Regular payment | Regular payment | Regular payment |

MHWs=Mental Health Workers; PSWs=Peer Support Workers

° Information provided by research workers at study sites
