# Supplementary material for: Mental health workers’ perspectives on peer support in high-, middle- and low income settings: a focus group study
Source: BMC Psychiatry. 2022 Sep 10;22:604. doi: 10.1186/s12888-022-04206-5 (PMC9464408; doi:10.1186/s12888-022-04206-5)
Supplement: Supplementary file 2 — Additional file 2. [file 12888_2022_4206_MOESM2_ESM.docx]

**Additional file 2: Focus group guide**

| Topic | Questions |
| --- | --- |
| 1. Collaboration | What is important for you when working with an UPSIDES Peer Support Worker (PSW)?  Additional questions:   - *In your opinion, what are the most important duties and responsibilities for PSWs?* - *Where do you see the opportunities of working together with a PSW? (for you, your team, the organization)* - *So far, we have spoken about the duties and responsibilities of PSWs. From your perspective, what are the main differences regarding the distribution of tasks between you and the PSW?* - *What could be challenges in working with a PSW?* - *How would you know that the collaboration with a PSW is working well?* |
| 1. Organizational and team culture | As an employee you know [name of organization] very well. In your opinion how well does UPSIDES Peer Support fit to the conditions within the organization?  Additional questions:   - *In your opinion how well do PSWs fit in your team/ your daily work process?* - *Do you expect that UPSIDES Peer Support in [name of organization] is going to work well? Why/ why not?* - *How do you think the work of the PSW will be accommodated by the team?* |
| 1. Support | What do you need in order to make the collaboration with the PSW to work well?  Additional questions:   - How well prepared do you feel to work with a PSW? - From whom would you like to receive support from for a successful collaboration with a PSW? |
