# Supplementary material for: Mental health workers’ perspectives on peer support in high-, middle- and low income settings: a focus group study
Source: BMC Psychiatry. 2022 Sep 10;22:604. doi: 10.1186/s12888-022-04206-5 (PMC9464408; doi:10.1186/s12888-022-04206-5)
Supplement: Supplementary file 3 — Additional file 3. [file 12888_2022_4206_MOESM3_ESM.docx]

**Additional file 3: Participants’ quotes**

Quotes are labelled according to the study site (Be’er Sheva (BGU), Butabika (BU), Dar es Salaam (DS), Ahmedabad (AD), Hamburg (UKE) and Ulm/Guenzburg (Ulm)) and the transcript chapter.

**1. Expected benefits from peer support**

“Live positively with mental illness and also to take care of themselves in that even when they leave the hospital, they can also be home and be very productive, other than being dependent on people around them or thinking they can only survive on the hospital as most patients believe” (BU 11)

“It comes from this idea to say, you have a shared experience and you make use of it and you get into a real contact and a real conversation and get to know each other and see what new possibilities I have. What do I actually want for my life? How can I get there?” (UKE 57)

“Earlier we used to advise the patient that you should do this or that. In contrast, a PSW makes the patient understand that they have also been affected by this disease and have recovered and through what criteria they’ve come out of it, which varies for everyone, for someone its yoga, or sharing your feelings. In this way, the PSW provide support to the patients and their relatives: ‘I’m here for you, I support you, tell me what is going on, and what do you want to do such that you improve?’, in contrast to advice” (AD 7)

PSW use “a different language than staff members (…) can reach service users on a different or common sense level and not that much on a professional one (…) PSW are allowed to say things directly and frankly because it’s more on an eye-to-eye level” (Ulm 86)

“Because it often comes from a different level, it may also have more credibility or it may simply have an ease where the other person doesn't feel bully or treated from above, but where he feels that this is more of a friendship, now come on, let's go” (Ulm 90)

PSWs “can easily explain how an experience is and how to live positively more than a mere mental health worker who has never experienced that” (BU 23)

“And I think to myself, the contact is simply really even closer and closer and closer” (UKE 186)

“Kind of ‘door opener’ for very skeptical patients with strong reservations against psychiatry, PSW can share their experiences and allay fears (…) because in acute situation there is often mistrust against staff” (Ulm 108)

“Let's say, in a group I am facilitating, so we'll say the same sentence. It will sound different if I or my partner say the same thing. Hmm… it's not always that dramatic, it doesn't always make a difference, but there are sentences, that if she says and if I say (makes a difference, SK)” (BGU 97)

Supporting service users through (in)formal arrangements

“They should be able to give evidence of a safety system which can’t provoke the disease or a person to relapse, for example there are some patients who use alcohol or substances which interfere with their medication so they should be able to advise them to change their life style” (DS 12)

“We will tell them about the side effects of the medicine but the peer support worker will be an advocate to give evidence of the side effects in using the medicine and what happened when he didn’t use medication. I will base on the profession part and for them it will (be) evidence based” (DS 27)

“(…)but they are not able to establish socially and economically so they get mentally reversed so for that if PSV guides them regarding vocational earning source like in (name of hospital welfare scheme), vocational day care, so many patients go there, so that population can increase, so accessing earning source can be advised as well“ (AD 137)

“If we can educate PSV about benefits that can be accessed for those with mental disabilities, I’ve seen maximum patients who roam around asking for eligibility of reservation these small guidance which usually takes the patients’ time, other than their existing skills if PSVs can do this as well, what benefits exist, to make certificate which documents are required for making certificate, governments’ requirements for little that can be helped and PSV can do this as well“ (AD 66)

“In my opinion, the current PSWs work seems to be a lot going somewhere, in wards, asking questions to patients, trying to understand them. Explaining rules and regulations is the current work. If we can go to a level of, for example the entry point to, in the beginning entering the role of observing them, knowing who’s their relatives, education level, taking initiative and talking to them, trying to understand the problem through the patient’s relatives, even if they can stay with one patient, that’s good as well” (AD 19)

“Not much but information related to psychiatrists, psychologists and social workers so that it’s easier during the registration process. See if they’re coming to the hospital then they’ll require some documents, now during admission process if the relatives say that, sometimes around 3-4 hrs in the admission process that they don’t have (name of identification document). So they realised that patient does not have it at this stage. Instead of that if the PSV can observe that a patient seems like they might get admitted, then the required documents and situations, what to talk to the doctor regarding symptoms, relatives are needed, these things can be made to understand” (AD 27)

“There (are) rules for particular regulations in every district so that should be made aware to patient because a lot of times we hurry up and complete the assessment and IQ test and then it is brought to notice from the first window that the person is from (name of another district), so if this is already known, problems will not be there” (AD 84)

Supporting service users in their communities: bridge building

“I think the main thing PSV should realise is that they are the link between hospital and the patient *(everyone nods yes)*. Neither are they hospital nor are they patient, when they come to take treatment that time they are patient, other than that they are link between hospital and patient (…) that is what should be developed and PSV should feel that her/his role is for making the patient get in touch, not forcefully, with the hospital services” (AD 117)

“The peer support worker should also be an ambassador to the service users, he/she should educate them and also engage those people who are close to the service user” (DS 18)

“So there will be an opportunity for me to understand a culture so that when am going to a specific community that have specific beliefs and all that, and put these things together to make sure that this community accepts us and then we are able to infiltrate the community” (BU 27)

“Because the language used is the official language, so I know that all of them will be familiar, language could have been a barrier if we were using English language or medical terms but if you are using a layman language then it will be understood to us and themselves” (DS 54)

“Will be listened to more than the health workers because they have gone through a similar experience which the other people are facing so they will be able to disseminate more information which will have an impact on the community than what it would be (…) they will be able to change the attitude towards mental health in the community” (BU 19)

Benefits for the PSW

PSWs learn that “they are useful in a way that they can be able to support the community so they also have to look after themselves very well to make sure that they are stable” (BU 19).

“Then it will also motivate other patients to how important it is to be economically independent, how can you do it. If we have developed these scopes in them [PSV], only then will he be able to develop these scopes in other patients. This is my thinking, whether it is possible or not” (AD 244)

“It will be a great benefit to them because we will make follow upon them so we are expecting that the number of relapses will decrease, they will be using medication and will be performing their tasks independently. We will also engage them in different activities such as sewing clothes, selling vegetables and this will make people in the community to realize that a person with a mental illness can also resume to perform his/her daily activities” (DS 25)

“PSW will benefit from us so I think our opportunity is to give them guidance, for example if they face a challenge with the service user they should come to seek advice from us. So I think that they will benefit more from us” (DS 20)

Benefits for the mental health services

„To me that is inspiration, to see a woman who went through so much, still going through so much, and still there is something very powerful about her, very strong“ (BGU 171)

“It’s a two-way opportunity in here. Working with a PSW helps a mental health worker to learn about the challenges in terms of treatment and recovery” (BU 27)

“There is a very good atmosphere among the entire staff. It is enriching, it makes it better. Such Upsides groups will be wonderful, in my opinion, both to the service users themselves, as well as to the consumers-as-providers. It's a place of joint empowerment for everybody. It's a natural matching-up, that's the feeling” (BGU 113)

“They will increase manpower in providing mental health services, somehow they are helping us in our work” (DS 77)

“A little bit of motivation, a little bit of activation, maybe serving as a positive role model and exchange of experiences. These things are often neglected because staff members are usually very busy” (Ulm 102)

**2. Challenges and concerns about peer support**

“I think it is complex. Because we all work according to how we experienced it. And in my staff, I really hear all the voices. Because there are those who will say that once there are consumers-as-providers, it means that we have to maintain it more, and we have to keep putting out fires constantly, as it were. And there are those who would say, you realize how amazing it is, that there is someone who really, truly identifies with you? Knowing what depression is, that you feel it in your entire body? I can really hear both voices, and I also believe that in the field, both voices exist” (BGU 114)

Negative effects on service users

Negative role model

“I also find it a bit difficult to find the right person, because the patients become really good again here, they usually return to their jobs normally, they are then back on the job market, they are employed full-time. They don't necessarily want to identify with psychiatry now either. And then the question is, who will be (a PSW)? (PSW) are often people who can't work full time, who are not so resilient and it was also a bit of university experience, we got the first one we had, he was just a bit older, he was very chronic, he couldn't work for a long time, he did it as a 450€ job and that was not really a role model for our young patients. That was more like that, okay, that's me now, maybe in 20 years and you have to be very careful who you find there” (Ulm 127).

“We had (PSW) who had been fully chronic (telling service users:) retirement is quite good, you can forget about working, it won't work anyway and about the medication, yes, but look at how I look, that's how you'll become” (Ulm 237)

“We have many incidences where many of them relapse because they snap out of their medicines, as they assume they are part of the hospital staff; and this affects them in their recovery process. This demoralizes those that have been seeing them as role models” (BU 39)

“But let's say, for example, one went through traumas due to a difficult divorce, the service user. And then she gets an instructor who also had a difficult divorce with traumas of violence. The instructor is sharing, innocently, they talk a little, and to her it brings up the traumas” (BGU 73).

Knowledge, skills and training

“The problem is that ward (X) in particular is a very, very sophisticated therapy concept, tailored to first contact. (…) and then I have a bit of a problem when someone who doesn't know our concept, who doesn't know what makes us tick, what we're up to, comes in from the outside (…) We don't know anything about him either, and that's a very delicate structure” (Ulm 136)

“The other challenge which I see and it might be with some of the peer support workers because there is the issue of providing education, providing education requires someone who has a good understanding of mental illness because there are some questions which require professional response” (DS 37)

“The peer support workers should be educated that they should not give false hopes to the service users who they are providing support to, other service users might think that you can take medication for two months and fully recover and stops using the medicine, then the peer support worker tells him/her that it possible, the person can do that and after a while he/she starts to experience the disease symptoms and will say that these people have come to lie to us, so they should be told to stick to the reality of the problem” (DS 38)

“If (PSW) states, that medication has no benefit, and (other things) helped me more, that is of course not very helpful. The same with drugs: When a (PSW) says, it's ok if you smoke pot again from time to time. It’s difficult to come to an agreement with what we represent, it doesn’t fit then” (Ulm 49)

„As I also said, we are so careful in one-on-one conversations, always bring in a lot of knowledge and experience and (we?) are so careful with every single person and they just go into a one-on-one conversation after a short training, and who knows what they are talking about with them. And we have no control over that at all“ (Ulm 125)

„For example, our psychotherapists in training, right? They have somehow studied for five years and then they really are, everything has to be supervised, they are not allowed to do anything on their own, we are so super careful and then after ten sessions a peer comes and is allowed to do everything on his own. Yes, I think that is a difficult point“ (Ulm 126)

“Many times they will come because of the motivation, because they want to do something. Because they felt that this is work that they can do. But the question is whether they went through a process. Not only with regard to their rehabilitation, also with regard to the understanding of what motivates them and how have you progressed with your rehabilitation” (BGU 150)

Negative effects on PSW

“I do hear, that's the truth, both sides, from the staff. Either it can greatly help and give some sense of partnership to the service-user, or identification. But it can sometimes also be another burden of some type” (BGU 129)

“That somehow decompensated him and I know that he was going to hospital one week later. (…) well, (peer support work) should also be something supportive for the peer and something that promotes self-esteem and not become a burden and I had the feeling that (PSW) put himself through too much and in the end, it was a burden for him too” (Ulm 259)

“I just want to add to this point that, currently the major issue that we have with this PSW structure is that there is no exit point. The people who are working for many years now, they are here only (…) yes, they seem to have stuck here” (AD 303)

“Exceeding the scope that a peer companion can provide" (UKE 17)

Negative effects on MHW teams and institutions

“They just go into a one-on-one conversation after a short training and who knows what they are talking about with them. And we have no control over that at all. Of course, this always happens when patients talk to each other, e.g. not taking medications anymore. But I just think that if it is an “official” service then, this is just not…it has no quality and we have to control that in any case and I think it is very important that they really get sufficient training and supervision” (Ulm 125)

“Sometimes a (PSW) might not be able to answer the questions (from service users) so the service users might lose trust with you” (DS 37)

“Because you sometimes maintain both the service-user, as well as the complexities that come up for the consumer but it can sometimes also be another burden of some type” (BGU 129)

**3. PSWs’ roles and boundaries**

(Need for) clarity of PSW’ roles

“It’s important to know their limits because sometimes they might cross over to do roles that are not intended within the domain of peer support and might go further to do the medical work” (BU 40)

“I: Do you think the presence of the peer support workers will take up your roles?

P: No” (DS 33)

“PSV is PSV, as a peer support group member, no one looks at them as a professional personnel anywhere in the world. And if that happens, then well and good” (AD 425)

“It's important to clarify the roles. So, in the treatment setting, what is the role of the PSW? Well, especially in comparison to the team. So, it's more of a mediating function, is it something completely different that's left out? Or is it rather someone who is on the same level as our visitors or patients and how, yes, what are the structures there? (…) how is that defined, or is it defined by each department according to its own criteria?” (Ulm 25)

“Then an ambiguity like this arises or the structures become watered down like this. And especially in our area or in the areas in which I work, structure is something really, really important” (Ulm 29)

“Neither are they hospital nor are they patient, when they come to take treatment that time, they are patient, other than that they are link between hospital and patient” (AD 109)

“If you take a hard look at the peer concept, then you have to say that we have nothing to do with the treatments and then say, okay, go to your therapist now and when you are healthy again, come back” (Ulm 318)

“But the important thing is that if they (PSWs) are also considered professionals then the peer feeling will go away” (AD 429)

Acceptance of role diffusions

“Because regarding the role, there is no difference among the instructors, a consumer-as-provider or not. The role is the same role. There is no change here in the role definition – the role is rehabilitation instructor and there is no difference here” (BGU 171)

“There is no difference between someone who is a professional employee and someone who is a consumer-as-provider (…) we all come from a certain life experience, with certain difficulties, that we encounter, facing the service-users and we really all work on it. With him, certain problems will come up, and with him, certain problems will come up” (BGU 179)

“How important it is to work as a team (…) they don't share, and feel that they know everything. That's also something I have encountered. That you, as a case management coordinator, ‘you don't know what I experienced. You don't know what it is. I know more’” (BGU 73)

“In our organization, there are places in which it is a designated role and the role definition has to be discussed” (BGU 167)

“New” vs. “established” PSW roles

B1: “What irritates me is also () I can already see from the offer that it is a definite competition.

I: It's not supposed to be there.

B1: It's not supposed to be that, I already said that, we talked about it a lot. But the feeling is just there. Where is there a difference?” (UKE 35)

**4. Team collaboration and PSWs’ position in mental health services**

“We do not have to shift our tasks to them; we are there to work together with them and look at the areas where they can ably support our services” (BU 33)

“There is readiness because they will increase manpower in providing mental health services, somehow they are helping us in our work” (DS 77)

“We will be providing technical advice and mentorship to them when we experience that there are shortcomings” (DS 31)

“We are ready by 100% because our role is to care for them, the important thing is to be empowered and work closely with them” (DS 76)

“We have to give proper guidance to them as well. Taking into consideration their education background, working background before the onset of the illness, current opportunity in that particular field. Hence, we should encourage them for their personal growth” (AD 300)

“And challenges will keep coming, day to day while working with new patients something will come up that they will face difficulty like what to do now, I don’t understand anything of this, so at that time other staff members can coordinate so PSV can get timely help and guidance” (AD 147)

PSW as “equal” partners

“There is no difference between someone who is a professional employee and someone who is a consumer-as-provider. The atmosphere is that everyone is equal. There is love, there is happiness in the togetherness, there is connection. There is a very good atmosphere among the entire staff. On the contrary, it is enriching, it makes it better” (BGU 113)

“The fact that I really trust him (PSW), that if he is now meeting with the service-user, he shares it with me and he is the eyes for that day, and to see what's happening with the service-user. Revealed intentions means that if I see something that is very important about the service-user, to share it with the consumer-as-provider, and not from a place of patronizing, that I know and you have to do, but to really share, since he is a partner, and we are both partners in accompanying that service-user” (BGU 70)

“We are together within the process, that I am not above, that not only I know, and not only he knows, and despite his knowledge and experience, he is involved in treatment. He is touching a different person, whose experience may not be the same experience. So for me, that's what's important throughout the process. We have to be together in this, and to understand this journey together” (BGU 91)

“Ok. Slowly, slowly, until we learn the sensitivities here, but you are capable. And that is a kind of strong backing that they need to hear. Not to say "No, no, no. There is difficulty, so goodbye" (BGU 66)

“The flexibility factor is built-in, even before the trend of the consumer-as-provider. It's something from the service I work at that was always there. And I think that it's something that always helps, even in this context. Because the flexibility is not because you are a consumer-as-provider, but a person. And every worker here has his need and you are very much aware of the special needs of everybody” (BGU 133)

“It's possible that for one person it would suit him perfectly and would make him very happy, and that to another it would be like insulting to his ego, "Why are you giving me someone me who is less?" (BGU 38)

“But I think there is still a stigma among the staff people. I see it a bit” (BGU 114)

“There were also animosities in the beginning. ‘Oh, so now I have to be an alcoholic, too, or what?’ which I think is empathetic. And meanwhile it's growing together really well, because we notice that we complement each other really well” (UKE 91)

Collaboration as (controlled) trust and commitment

“So, this also implies a certain commitment, if I want to work here as a peer support worker, then I also need a commitment to the institution or ward where I will be, and that means that there must be an understanding of what belongs to the spectrum and these topics come up for me when I ask the question- does it fit into the concept and is it imaginable? I would say that for me it is conditional if it is clarified and if the wards it concerns are also included, what is important to clarify or in this clarification process. Is it now a team member or is it someone who comes from outside, perhaps as a kind of pastor, as it were, and then speaks about the needs outside of the times. But that, I think, must be clear, does it fit into the concept here? Then I would say, yes, it does, when it's clarified” (Ulm 218)

“One or three PSW coming to our ward, which you get to know over time and then, yes, it grows. We will also grow together to a certain extent. If it is always someone else, then I still consider it difficult” (Ulm 214)

“Regarding collaboration, it is very important to have good exchange and open discussion. I don’t want to have the feeling that PSW are doing their own thing which we do not know anything about it. Openness and feed-back is very very important” (Ulm 37)

“Well, if we can learn from mistakes in the past, then it will work well (…) we can learn more from hospitals where it works well” (Ulm 237)
